# Assessing Patient-Reported Satisfaction With Care and Documentation Time in Primary Care Through AI-Driven Automatic Clinical Note Generation: Protocol for a Proof-of-Concept Study

**DOI:** 10.2196/66232

**Published:** 2025-04-07

**Authors:** Josep Vidal-Alaball, Carlos Alonso, Daniel Hugo Heinisch, Alberto Castaño, Encarna Sánchez-Freire, María Luisa Benito Serrano, Carla Ferrer Pascual, Ignacio Menacho, Ruthy Acosta-Rojas, Odda Cardona Gubert, Rosa Farrés Creus, Joan Armengol Alegre, Carles Martínez Querol, Marina Moreno-Martinez, Mercè Gonfaus Font, Silvia Narejos, Anna Gomez-Fernandez

**Affiliations:** 1 Research and Innovation Unit Gerència d'Atenció Primària i a la Comunitat de la Catalunya Central Institut Català de la Salut Manresa Spain; 2 Intelligence for Primary Care Research Group Fundació Institut Universitari per a la Recerca a l'Atenció Primària de Salut Jordi Gol i Gurina Manresa Spain; 3 Department of Medicine Faculty of Medicine University of Vic-Central University of Catalonia Vic Spain; 4 Recog Analytics Madrid Spain; 5 Artés Primary Care Team Gerència d'Atenció Primària i a la Comunitat de la Catalunya Central Institut Català de la Salut Artés Spain; 6 Les Corts Primary Care Centre Consorci d'Atenció Primària de Salut Barcelona Esquerra Barcelona Spain; 7 Transversal Primary Care Research Group Institut d'Investigacions Biomèdiques August Pi i Sunyer Barcelona Spain; 8 Centelles Primary Care Centre Centelles Spain; 9 Súria Primary Care Team Gerència d'Atenció Primària i a la Comunitat de la Catalunya Central Institut Català de la Salut Súria Spain; 10 Sallent Primary Care Team Gerència d'Atenció Primària i a la Comunitat de la Catalunya Central Institut Català de la Salut Sallent Spain; 11 Center for the Integration of Medicine and Innovative Technologies Fundación Leitat Barcelona Spain

**Keywords:** primary health care, patient satisfaction, artificial intelligence, medical records systems, computerized, patient-centered care

## Abstract

**Background:**

Relisten is an artificial intelligence (AI)–based software developed by Recog Analytics that improves patient care by facilitating more natural interactions between health care professionals and patients. This tool extracts relevant information from recorded conversations, structuring it in the medical record, and sending it to the Health Information System after the professional’s approval. This approach allows professionals to focus on the patient without the need to perform clinical documentation tasks.

**Objective:**

This study aims to evaluate patient-reported satisfaction and perceived quality of care, assess health care professionals’ satisfaction with the care provided, and measure the time spent on entering records into the electronic medical record using this AI-powered solution.

**Methods:**

This proof-of-concept (PoC) study is conducted as a multicenter trial with the participation of several health care professionals (nurses and physicians) in primary care centers (CAPs). The key outcome measures include (1) patient-reported quality of care (evaluated through anonymous surveys), (2) health care professionals’ satisfaction with the care provided (assessed through surveys and structured interviews), and (3) time saved on clinical documentation (determined by comparing the time spent manually writing notes versus reviewing and correcting AI-generated notes). Statistical analyses will be performed for each objective, using independent sample comparison tests according to normality evaluated with the Kolmogorov-Smirnov test and Lilliefors correction. Stratified statistical tests will also be performed to consider the variance between professionals.

**Results:**

The protocol has been developed using the SPIRIT (Standard Protocol Items: Recommendations for Interventional Trials) checklist. Recruitment began in July 2024, and as of November 2024, a total of 318 patients have been enrolled. Recruitment is expected to be completed by March 2025. Data analysis will take place between April and May 2025, with results expected to be published in June 2025.

**Conclusions:**

We expect an improvement in the perceived quality of care reported by patients and a significant reduction in the time spent taking clinical notes, with a saving of at least 30 seconds per visit. Although a high quality of the notes generated is expected, it is uncertain whether a significant improvement over the control group, which is already expected to have high-quality notes, will be demonstrated.

**Trial Registration:**

ClinicalTrials.gov NCT06618092; https://clinicaltrials.gov/study/NCT06618092

**International Registered Report Identifier (IRRID):**

DERR1-10.2196/66232

## Introduction

### Background

In the medical field, medical record writing is an essential task that requires time and accuracy on the part of health care professionals. The medical record, which includes the patient’s medical history, any symptoms they have had, treatments performed, and other relevant details, is a critical component in making appropriate medical decisions and ongoing patient follow-up [1].

In the modern health care context, there has been a transition to the digitization of these records, giving rise to the concept of the electronic medical record (EMR). An EMR is the electronic representation of a patient’s medical record, created and maintained by health care professionals. This digital approach has not only revolutionized the way medical information is stored and accessed but has also improved the efficiency of medical care by facilitating the retrieval of relevant data at the point of care [2,3]. EMRs provide a centralized platform for medical information management, allowing for more accurate tracking and more coordinated care [4].

Traditionally, health care professionals have spent a significant amount of time writing medical records [5], which can affect the efficiency and quality of care they provide. This manual task is not only time-consuming but can also lead to documentation errors, omissions, or inconsistencies in the information recorded. A descriptive study published in the *Annals of Internal Medicine* in 2020 conducted a detailed assessment of the amount of time doctors spent using EMRs during outpatient visits. In this study, which included comparisons of 1482 doctors, it was found that doctors were actively using EMRs for 43% of the total time they were online [6]. It has also been shown that all this can contribute to an increase in burnout among professionals [7,8].

In recent years, the field of artificial intelligence (AI) has experienced significant advances in natural language processing and speech recognition [9,10]. These advances have enabled the creation of automated tools and systems that can accurately and efficiently transform speech into text. In the health care setting, this technology has the potential to streamline and improve the writing of medical records, freeing up time for professionals to focus on direct patient care [11,12]. However, this technology was necessary but not sufficient, and it was not until the advent of generative AI that a key part of the process could be completed to obtain sufficient quality for practical use [13,14].

Another important aspect is the issue of data privacy and security [15]. The implementation of AI systems in clinical practice must comply with strict regulations regarding the protection of patient health data. This includes ensuring the security of data transmission and storage, as well as compliance with data privacy regulations, and the General Data Protection Regulation [16]. In the case of AI use, these requirements must go further, given the additional risks of training models with data in health care, which in the worst cases can result in data hacking or the inclusion of strong biases in the algorithms [17,18].

Several companies and academic institutions have developed prototypes and pilot systems for the automated writing of medical records from the doctor-patient conversation in English (Nuance DAX, Amazon HealthScribe). These tools use advanced algorithms and AI models trained on large amounts of clinical data to extract and record relevant information [19].

### Relisten

In this context, the Spanish company Recog Analytics has developed Relisten [20], an automated clinical note-writing system designed to handle real-world consultations, supporting multiple languages and offering integration with EMRs.

Through a natural conversation between the health care professional and the patient, this AI-powered tool uses recordings to extract relevant fields corresponding to key sections of the medical history in a structured way and then send them to the Health Information System (after correction and approval by the health care professional). A detailed description of the algorithm’s architecture can be found in Multimedia Appendix 1, providing technical insights into its design and functionality.

## Methods

### Study Objectives

This study has the following objectives: (1) to evaluate the perceived quality of care reported by patients with the use of an AI-powered solution in the consultation, measured through anonymous satisfaction questionnaires at the end of each consultation (Multimedia Appendix 2); (2) to assess the satisfaction of health care personnel with the care provided in consultations, measured using an anonymous satisfaction survey at the end of the study (Multimedia Appendix 3) and a structured interview; and (3) to assess the time spent by health care personnel on entering records in the EMR, measured by statistical tests comparing consultations with and without the tool.

### Overview, Design, and Hypotheses of Study

A proof-of-concept (PoC) analysis will be carried out in the context of a multicenter study, where several health professionals (nurses and physicians) from different primary care centers (CAPs) will use the AI-powered tool in consultations (under informed consent of the patient) and will survey patients and the professionals themselves.

The project is structured in three main phases: (1) preparation: this includes the demonstration and initial training of the participating professionals, the preparation of the technological infrastructure (microphones), and the coordination for carrying out the surveys, with an approximate duration of 2 weeks; (2) development: this consists of the use of an AI-powered solution during consultations, as well as data collection, including audios, clinical notes, and survey results, lasting approximately 20 weeks; and (3) data analysis and presentation of results: this phase involves the processing and analysis of the data collected, followed by the presentation of the results obtained, and lasts approximately 2 weeks.

The measurement of the objectives will be carried out using the following methods: (1) perceived quality of care as reported by patients using an anonymous patient survey after each consultation, in a patient-blinded study (patients are not told beforehand whether the AI-powered tool has been used in the consultation; this fact is outlined by a graphic mark in the surveys in which the tool has been used); (2) satisfaction of health care personnel: an anonymous survey will be conducted at the end of the study. This will qualitatively evaluate the tool’s impact on consultations, focusing on aspects such as its general usefulness, ease of use, the quality of notes generated, its perceived ability to improve attention during consultations, and the extent to which it saves time. In addition, the study will incorporate structured interviews with primary care health care professionals (nurses and physicians) to explore their experiences with the tool in depth. The interviews will include questions about the above concepts, as well as at least an additional request to the health care professional to identify limitations and improvements to the tool. (3) Time spent entering information into the EMR. To determine the magnitude of savings, 2 measurements taken during the PoC will be compared: the time the health care professional spends typing in the consultation and the time the health care professional spends reviewing and correcting notes generated by the AI-powered tool.

**Figure 1:**
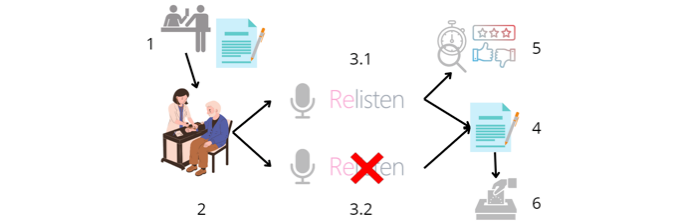
Steps involved in the study workflow.

Figure 1 facilitates the visualization of the development of the study in consultations.

The steps mentioned in Figure 1 include:

1. Patient consent at reception: the patient arrives at the CAP and is attended to at the reception desk, where he or she is given the information sheet and the informed consent form, which he/she will return signed if he/she agrees to participate in the study.
2. Consultation begins: the patient arrives for consultation and the doctor confirms their participation.
3. (3.1) Use of Relisten: the consultation is carried out, alternating between consecutive patients (simple randomization). Recording and using the AI-powered tool to provide full patient care. In this mode, the health professional does not take notes during the consultation and proceeds to step 5.
4. (3.2) Standard documentation: recording it and writing it at the same time in the electronic record as usual. In this mode the AI-powered tool is only used as a consultation recorder to calculate the typing time of the health care professional, using these consultations as a control group.
5. Note review or writing: the doctor hands the survey to the patient and says goodbye.
6. Satisfaction surveys: the doctor reviews the notes generated by the AI-powered tool, recording the time required for that task directly on the tool’s website, copies and pastes them into the electronic record, and rates the notes generated by the tool for that consultation.
7. Anonymous survey submission: the patient deposits the completed survey anonymously in the box provided.

The study team hypothesizes that the use of an AI-powered tool improves the perceived quality of care by allowing health care professionals to spend more time interacting directly with patients and less time on clinical documentation. This translates into more patient-centered care and a measurable improvement in both patient and staff satisfaction.

### Setting of the Study

The study population will be made up of patients who are visited in person at CAPs in Catalonia, both in emergency and control consultations, whose professionals (nurses and physicians) participate in the study. The participating primary care centers are CAP Amposta, CAP Centelles, CAP Artés, CAP Sallent, CAP Súria, and the Consorci d’Atenció Primària de Salut Barcelona Esquerra.

Given the nature of the tool and its applicability to most of the practice settings, there will be no previous selection of patients, but any patient who comes for consultation with the professionals participating in the study will be eligible.

Participants eligible for inclusion in the study must have provided written informed consent, be involved in face-to-face medical or nursing consultations conducted in Catalan or Spanish, and be aged 18 years or older.

Participants will be excluded from the study if they are unable to understand the nature of the study, lack fluency in Catalan or Spanish, do not provide consent to be recorded, or if any technical issues, such as internet service downtime, compromise the recording process.

The study included patients in unscheduled visits (emergency), first visits (with history taking), and follow-up for chronic disease.

The SPIRIT guidelines were followed when designing the research (Multimedia Appendix 4) [21].

### Sample Size and Randomization

To calculate the sample size, the 2 objectives that require such calculation are considered:

1. For the patient satisfaction surveys, proposed on a Likert scale from 1 to 5, we are faced with the problem of comparing independent samples. Assuming normality in the difference of the samples (a hypothesis that will be verified during the statistical tests), the formula for the determination of the required sample size is the following: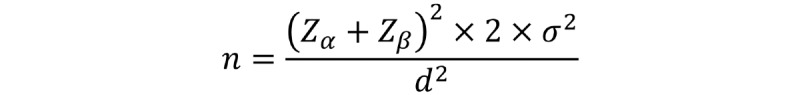  Where Z_α_ and Z_β_ represent the Z-table values for confidence level and test power, respectively, sigma is the SD of the (estimated) differences, and d is the effect size to be measured. For this study, standard values for α=.05 (95% confidence) and β=.8 (80% test strength) are selected, σ=1 is estimated as an extreme case to ensure that the results will be statistically significant, and d=0.25, as the minimum improvement we want to demonstrate. The result is n=395.21 for each group, so we set the number of patients to be analyzed to 400 with the AI-powered tool and 400 without the tool.
2. For time-saving, applying a similar procedure with the same standard values, σ=90 seconds and d=30 seconds, we obtain n=111.15 for each group, so we set the number of consultations to be analyzed to 120 with the AI-powered tool and 120 without the tool.
  Since the groups are the same and there is no interconnection between the measurement of both objectives, it is possible to use the same consultations for both, and therefore the total number of patients required will be the larger of the two above, resulting in n=800 (400 patients with the AI-powered tool and 400 without the tool).
  The randomization was carried out in a simple format, given the sufficient sample size and the fact that the aim is to validate the hypotheses at a general level, although for information purposes the study also shows intermediate results stratified by type of consultation (first or follow-up) or by a health professional.

### Data Collection

The data collection process involves the following:

1. Satisfaction surveys completed by patients at the end of the consultation (with the AI-powered tool as a test and without the tool as a control). The survey will be given to the patient on paper by the health professional inside the doctor’s office at the end of the visit. They will be collected anonymously in a box upon exiting the office, in a visible and accessible place. This involves single-blinded data collection (the patient does not know whether the AI-powered tool is being used, although the health care professional does). These questionnaires will be available in Catalan and Spanish.
2. Quality surveys at the end of each consultation by health care professionals, on a scale of 0-10 with the possibility of including open-ended comments, will be carried out directly through the AI-powered tool’s digital platform. These questionnaires will be available in Catalan and Spanish.
3. Satisfaction surveys completed by health care professionals at the end of the study, as well as interviews to better understand the impact and limitations in a qualitative manner.
4. Recordings of conversations between the patient and health care professional (Recog will delete the recordings at the end of the study).

These items align with study objectives and are detailed in the Multimedia Appendices 2 and 3.

Qualitative data will be collected using open-ended survey surveys for patients and health care professionals and structured interviews with professionals. These data will be analyzed using thematic analysis.

The study variables are mentioned in Textbox 1.

**Study variables.**

**Independent variables**

- Indicator of whether the artificial intelligence (AI)–powered solution has been used in the consultation (no typing during the consultation; yes or no)
- Identifier of the health care professional involved (user code stored in the AI-powered tool)

**Dependent variables**

- Results for each survey question, on a scale of 1-5.
- Time saved during consultations is measured by comparing the writing time in the 2 scenarios: one where doctors manually write notes during the consultation (non–AI-powered audios) and another where they review and correct notes generated by the AI-powered tool. For the non–AI-powered audios, investigators listen to the recordings and measure the exact time the professional spends writing. In AI-assisted consultations, the doctors use the platform to time their note corrections. By comparing these two measurements, the study aims to quantify the time-savings offered by the AI-powered tool, with each recording being analyzed individually for accuracy. This has been chosen because other forms of measurement (such as automatically measuring silences) may introduce greater biases (silences due to circumstances other than note taking) than human interpretation. Although logically it is not a perfect measure either, the savings impact is expected to be sufficiently significant for these random errors to have a very small impact in relative terms.
  In addition, health care professionals are asked to include, through the AI-powered tool’s digital platform, possibly relevant comments about the consultation they have just carried out, such as when a consultation has been carried out in multiple languages, with one participant speaking in Spanish and another in Catalan.

### Statistical Analysis

The main hypotheses of the study focus on evaluating the reported quality of care and saving time in the writing of clinical notes. To assess the improvement in reported quality of care, the normality of the sample will be checked using the Kolmogorov-Smirnov test with Lilliefors correction. Student *t* test for independent samples will be applied in the case of normality, or an equivalent nonparametric test otherwise. Key survey items, including patient-reported satisfaction with time spent, the attention received, and perceived care quality, as well as health care professional satisfaction with reduced administrative burden, ease of use, and note quality, will be analyzed to test for significant differences.

In terms of time savings, 2 aspects will be measured separately: the relative time (in percentage) that the professional spends writing notes during the consultation without using the AI-powered solution, and the relative time that the doctor spends reviewing the notes generated by the tool. Similarly, the normality of the sample will be checked using the Kolmogorov-Smirnov test with Lilliefors correction, and the Student *t* test or its nonparametric equivalent will be applied as appropriate.

Since the sample is not randomized, a sensitivity analysis will be conducted to account for potential selection bias. All statistical analyses will be performed using RStudio, considering a confidence level of 95% and a statistical power of 80%.

### Confidentiality

In this study, confidentiality will be rigorously protected by several procedures. The patient surveys, which will be conducted anonymously upon exiting the consultation, will be transcribed by the Recog staff into a Microsoft Excel file for subsequent analysis, without any identifying data. The audios collected during the consultations will be automatically stored in the AI-powered tool platform, guaranteeing their security by storing them in the S3 (Simple Storage Service) service of Amazon Web Services, with controlled access, encryption, and without identifiers that allow patients to be identified. The professionals’ comments will be stored in Amazon Web Services DynamoDB tables, also with controlled access, thus ensuring the confidentiality of all the information collected.

In no case is the objective of the study to train the algorithm with the data obtained, since the algorithm is already trained. The models applied are speech-to-text models, originally trained on multilingual datasets [22] and a large language model trained on generic datasets in multiple languages, public and private, not specified by the model developer. Notes are written in the project and no medical analysis is being performed by AI. The initial tests have shown the correct capability of the complete software for this task, which we seek to validate more extensively with this project. The initial stages of the algorithm use machine learning techniques, such as advanced speech-to-text models for transcription. The subsequent steps, including structuring the extracted information into relevant clinical fields, rely on a combination of rule-based processes, predefined templates, and generative AI models.

### Ethical Considerations

This study was approved by the University Institute for Primary Care Research Jordi Gol Health Care Ethics Committee (Code 3/286-P). The research adhered to institutional guidelines for studies involving human subjects, including those requiring the recording of patient interactions and subsequent analysis. Written informed consent will be obtained from all participants before their inclusion in the study. Participation was entirely voluntary. No monetary or material compensation was provided to participants. All participants will complete a written informed consent. Upon arrival at the CAP, the Admissions administration staff will offer the patient the chance to participate in the study. The participant will be given the information sheet attached to the informed consent form, where he or she will be informed about the purpose of the study and the handling of the data. If the patient authorizes his or her participation, he or she must complete and sign the informed consent form and give it to the clinical professional with whom the consultation is to take place.

## Results

This study was preregistered on ClinicalTrials.org. Recruitment began in July 2024, and as of November 2024, a total of 318 patients have been enrolled at participating CAPs. The recruitment process is proceeding more slowly than expected and to ensure sufficient sample size the recruitment period has been extended until March 2025. This adjustment enables comprehensive data collection without compromising the study’s objectives.

## Discussion

### Principal Findings

The implementation of AI-based technologies in health care has been a topic of growing interest in the last decade. These technologies promise to improve the efficiency and quality of health care, but their adoption depends largely on empirical evidence to support their benefits.

This study seeks to contribute to that evidence base by evaluating a specific tool that has the potential to alleviate one of the main sources of administrative burden for health care professionals: the writing of clinical notes. By freeing up time that would otherwise be spent on administrative tasks, an AI-powered tool could enable health care professionals to focus more on direct patient care, thereby improving the quality of care.

The results of the project will serve to validate the usefulness of an AI-powered solution in daily practice, from the perspectives of improving the perceived quality of care and saving professionals’ time, which could amount to more than an hour a day that could be invested in attending to more patients, promoting adherence with the same patients, or other value-added tasks.

If the result of the study is favorable, the tool is technically prepared to be integrated with IT systems, is scalable to thousands of queries in parallel and has been designed from the ground up with absolute priority to data security and privacy (the tool deletes queries minutes after processing to avoid being a target for cyberattacks; it is not a database, just a processor). It is therefore considered that its implementation may be feasible, the first step being to validate its capabilities in this study.

While our study is one of the first to evaluate the use of an automated clinical note-writing system based on AI algorithms in day-to-day clinical consultations in primary care, previous research has explored similar technologies in other medical specialties. For example, a 2024 study assessed the accuracy of AI-generated clinical notes using ChatGPT-4 (OpenAI), finding that such tools can produce notes comparable in quality to those taken by physicians [23]. In another study, the Permanente Medical Group (United States) implemented AI scribes for more than 10,000 physicians and found that this technology reduced documentation time, improved doctor-patient interactions, and achieved high satisfaction among both patients and physicians [24].

### Limitations of the Study

One of the main limitations of this study is that the sample is not randomized, which may introduce selection bias. To address this, a sensitivity analysis will be performed to ensure that any potential bias is minimized. Related to the sample size, while substantial for a PoC study with 800 participants, may still be considered relatively small.

Another important limitation is the variability introduced by the participation of different health professionals, who can generate a significant variance in the results obtained, both in patient satisfaction and in the time saved in the consultation. Although patients were blinded to the use of the tool, their reported satisfaction may still be influenced by other factors such as the health care professional’s behavior or consultation dynamics. To mitigate this effect, it has been decided to involve a reasonable number of professionals, seeking a balance between reducing variance and avoiding an excessive burden on participants, although this will still be a limiting factor.

Another possible bias is in the measurement of time saved. This is calculated as the difference between the time spent on a consultation without the AI-powered tool to write the notes and the time needed to review the notes generated by the tool in another consultation. However, there will always be an inherent bias, as it is not possible to reproduce the same consultation exactly, and the professional’s previous knowledge may influence how quick they are in carrying it out. In addition, the measurement of the time the professional spends writing may not be completely accurate. In addition, while we assess the time spent on writing clinical notes, another potential limitation lies in the time spent reviewing the notes generated by the tool. This measurement may be influenced by external factors, such as interruptions (eg, being called to the door, receiving phone calls, etc), which could result in prolonged review times without the actual time being spent on reviewing the notes. The start and end points of the review period are based on when the professional clicks “start review” and “finish review,” but interruptions could lead to inaccurate time recordings. In addition, another limitation is the risk that doctors rely too much on AI-generated notes without thoroughly reviewing them, which could lead to inaccuracies. Although doctors are instructed to verify the notes as soon as possible following the consultation to avoid this, individual differences in review diligence could have an impact on the results. Despite these challenges, an analysis of these errors has been performed and it has been concluded that their impact will be significantly lower than the effects that are intended to be measured, so the results, with the proposed sample, can be considered valid. However, if any extreme values are identified during the analysis, they will be excluded.

### Conclusion

We anticipate that patients participating in the study will perceive an improvement in the quality of care they receive and that there will be a significant reduction in the time spent taking clinical notes. Although we expect the generated notes to be of high quality, it remains uncertain whether a significant improvement over the control group, which is already expected to have high-quality notes, will be demonstrated. Ultimately, this proof-of-concept study seeks to explore the potential benefits of integrating AI in primary care settings.

## Appendix

Multimedia Appendix 1 Algorithm Architecture.

Multimedia Appendix 2 Health Professional Satisfaction Survey.

Multimedia Appendix 3 Patient Satisfaction Survey.

Multimedia Appendix 4 SPIRIT (Standard Protocol Items: Recommendations for Interventional Trials) checklist.

## Abbreviations

AI: artificial intelligence
CAP: primary care center
EMR: electronic medical record
PoC: proof of concept
S3: Simple Storage Service
SPIRIT: Standard Protocol Items: Recommendations for Interventional Trials

## Data Availability

The datasets used and/or analyzed during the current study will be available from the corresponding author on reasonable request.
