# Supplementary material for: Assessing Patient-Reported Satisfaction With Care and Documentation Time in Primary Care Through AI-Driven Automatic Clinical Note Generation: Protocol for a Proof-of-Concept Study
Source: JMIR Res Protoc. 2025 Apr 7;14:e66232. doi: 10.2196/66232 (PMC12012399; doi:10.2196/66232)

**Appendix I: Algorithm Architecture**

The AI tool used in this study employs a combination of advanced speech-to-text models and generative AI systems. The speech-to-text component is designed to handle multi-language inputs, while the generative AI system, combined with a template segregation, structures the extracted information into relevant fields for clinical documentation. The system does not retain or train on the study data, ensuring compliance with strict data privacy standards.

The main processing steps are:

- The entire audio file is sent to a speech to text model. Distance based correction mechanisms were implemented to improve recognition of clinical vocabulary.
- Several prepared LLMs calls are performed in parallel to retrieve information for different parts of the notes (history of present illness, background, plan…). The input for each call is a combination of a system prompt, a user prompt and the transcription output by the speech to text model.
- An additional LLM step was performed for each of the outputs from the previous steps to verify results and improve medical terminology.
- Final output is a structured object representing the clinical note divided into sections and individual fields with the relevant information extracted and corrected from the conversation.

Particular models and prompts are not shared due to IP limitations.


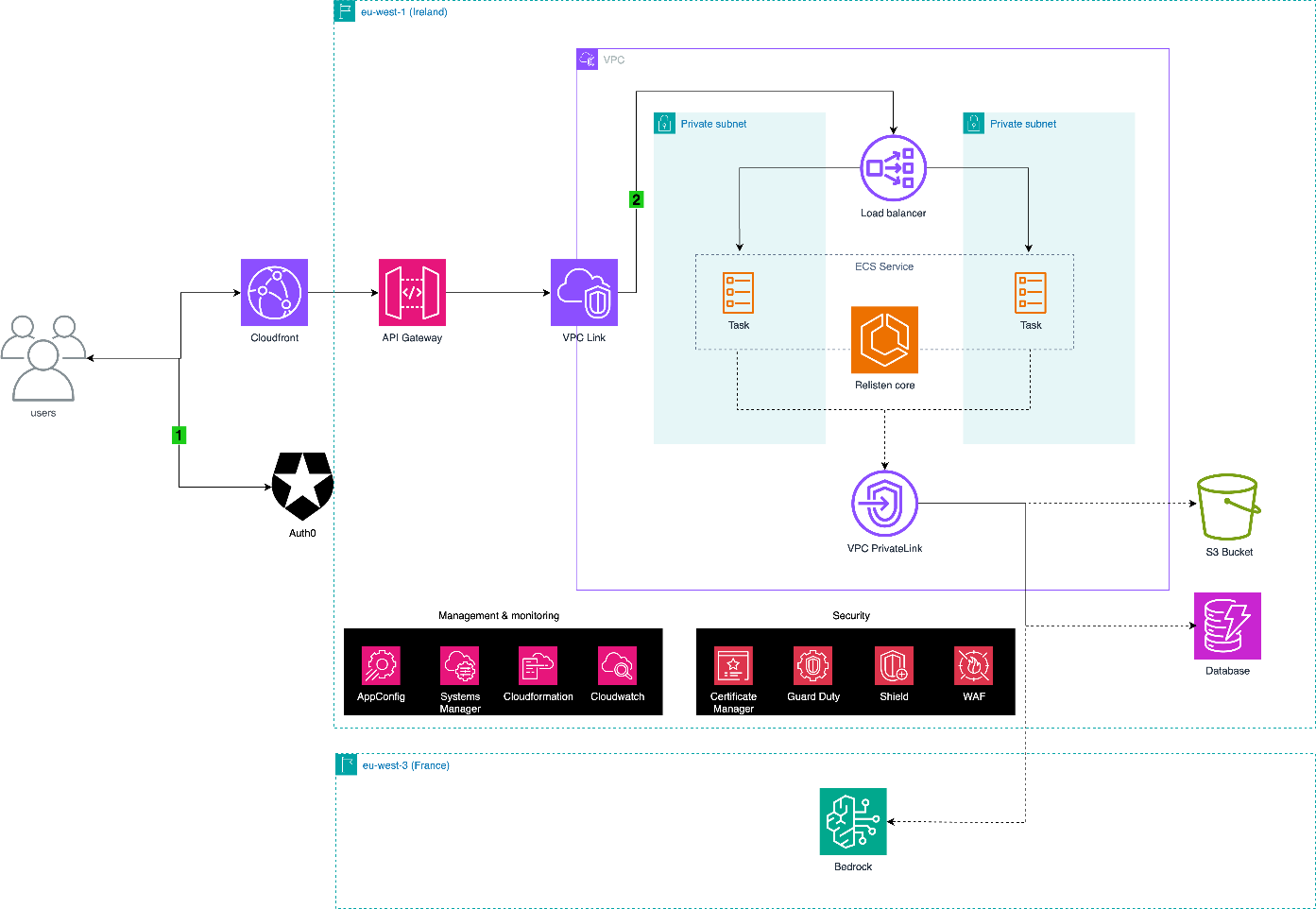

Supplement: Multimedia Appendix 1 [file resprot_v14i1e66232_app1.docx]
