# Supplementary material for: Assessing Patient-Reported Satisfaction With Care and Documentation Time in Primary Care Through AI-Driven Automatic Clinical Note Generation: Protocol for a Proof-of-Concept Study
Source: JMIR Res Protoc. 2025 Apr 7;14:e66232. doi: 10.2196/66232 (PMC12012399; doi:10.2196/66232)
Supplement: Multimedia Appendix 2 [file resprot_v14i1e66232_app2.docx]

**Appendix II: Health professional satisfaction survey**

| 1. **Rate the following Relisten aspects from 1 to 6**  \|  \| 1 (not at all) \| 2 \| 3 \| 4 \| 5 \| 6 (very much) \| \| --- \| --- \| --- \| --- \| --- \| --- \| --- \| \| The usefulness of the summary of the relevant fields of the medical record \|  \|  \|  \|  \|  \|  \| \| Understanding the summary of the fields relevant to the medical record \|  \|  \|  \|  \|  \|  \| \| Reducing the administrative burden \|  \|  \|  \|  \|  \|  \| \| Reducing the time spent on typing in the health information system \|  \|  \|  \|  \|  \|  \| \| Efficient medical data collection has been improved \|  \|  \|  \|  \|  \|  \|  1. **Were you able to pay attention to the patient during the entire visit?**  - Yes, much more - Yes, but not significantly - No, I have paid the same attention as before - Don't know / No answer  1. **Did you take notes during the visits?**  - No, I have not taken any notes - Yes, I have taken fewer notes, but I have still written down some information - The same as before - Don't know / No answer  1. **Did you have any incidents during the visit due to the information systems?**  - Yes, resolved by the technical service promptly - Yes, they were resolved after a few days - No - Don't know / No answer  1. **In general, how much interest do you have in using Relisten?**  \| 1 (no interest) \| 2 \| 3 \| 4 \| 5 \| 6 (high interest) \| \| --- \| --- \| --- \| --- \| --- \| --- \|  1. **On a scale of 1 to 6, how useful do you consider the use of an assistant in the visit in the following cases:**  \|  \| 1 (not at all) \| 2 \| 3 \| 4 \| 5 \| 6 (very much) \| Not applicable \| \| --- \| --- \| --- \| --- \| --- \| --- \| --- \| --- \| \| First visit \|  \|  \|  \|  \|  \|  \|  \| \| Chronic patient follow-up \|  \|  \|  \|  \|  \|  \|  \| \| Emergency visit (unscheduled) \|  \|  \|  \|  \|  \|  \|  \| |
| --- | --- | --- | --- | --- | --- | --- | --- | --- | --- | --- | --- | --- | --- | --- | --- | --- | --- | --- | --- | --- | --- | --- | --- | --- | --- | --- | --- | --- | --- | --- | --- | --- | --- | --- | --- | --- | --- | --- | --- | --- | --- | --- | --- | --- | --- | --- | --- | --- | --- | --- | --- | --- | --- | --- | --- | --- | --- | --- | --- | --- | --- | --- | --- | --- | --- | --- | --- | --- | --- | --- | --- | --- | --- | --- | --- | --- | --- | --- | --- | --- |

1. **In general, would you recommend the use of Relisten to other colleagues?**

| 1 (not at all) | 2 | 3 | 4 | 5 | 6 (very much) |
| --- | --- | --- | --- | --- | --- |
