# Supplementary material for: Assessing Patient-Reported Satisfaction With Care and Documentation Time in Primary Care Through AI-Driven Automatic Clinical Note Generation: Protocol for a Proof-of-Concept Study
Source: JMIR Res Protoc. 2025 Apr 7;14:e66232. doi: 10.2196/66232 (PMC12012399; doi:10.2196/66232)
Supplement: Multimedia Appendix 3 [file resprot_v14i1e66232_app3.docx]

**Appendix III: Patient satisfaction survey**

| 1. **Age** |
| --- |
| 18-24 years old |
| 25-34 years old |
| 35-44 years old |
| 45-54 years old |
| 55-69 years old |
| More than 70 years old |
| 2. **Sex** |
| Male |
| Female  Non-binary |
| I prefer not to say |
| 3. **Studies** |
| No formal education or qualification  Compulsory studies  Post-compulsory education  I prefer not to say |
| 4. **Work activity** |
| Full-time employee or self-employed  Part-time employee or self-employed |
| Student  Retired  Out of work / unemployed  Other |

**5. Has the doctor introduced himself/herself and greeted you cordially?**

Absolutely / Very much / Yes / Not very much / Not at all

**6. How do you feel about the time the doctor spent on you when treating you?**

Perfect / Very good / Good / Fair / Poor

**7. What do you think of the doctor's willingness to listen to you?**

Perfect / Very good / Good / Fair / Poor

**8. And what do you think about the willingness to take care of your health concerns?**

Perfect / Very good / Good / Fair / Poor

**9. Do you feel you are in good hands?**

Absolutely / Very much / Yes / Not very much / Not at all

**10. How do you rate the doctor's treatment and kindness towards you?**

Perfect / Very good / Good / Fair / Poor

**11. To what extent would you say that the professional who attended you was relaxed and had plenty of time to attend to you?**

Absolutely / Very much / Yes / Not very much / Not at all

**12. To what extent have you received more information about your problem from your doctor?**

Absolutely / Very much / Yes / Not very much / Not at all

**13. To what extent have you felt cared for and respected with regard to the confidentiality of your data?**

Absolutely / Very much / Yes / Not very much / Not at all

**14. If you use a medical aid, has it been explained to you, and have you been asked for permission to use it?**

Absolutely / Very much / Yes / Not very much / Not at all

**15. Additional comments / areas for improvement**

(optional free text field)
